# Effect of Bread Structure and In Vitro Oral Processing Methods in Bolus Disintegration and Glycemic Index

**DOI:** 10.3390/nu11092105

**Published:** 2019-09-04

**Authors:** Andrea Aleixandre, Yaiza Benavent-Gil, Cristina M. Rosell

**Affiliations:** Institute of Agrochemistry and Food Technology (IATA-CSIC), C/Agustin Escardino, 7, 46980 Paterna, Valencia, Spain (A.A.) (Y.B.-G.)

**Keywords:** bread, matrix structure, oral digestion, bolus particle size, glycemic index

## Abstract

The growing interest in controlling the glycemic index of starchy-rich food has encouraged research about the role of the physical structure of food. The aim of this research was to understand the impact of the structure and the in vitro oral processing methods on bolus behavior and starch hydrolysis of wheat bread. Two different bread structures (loaf bread and bread roll) were obtained using different shaping methods. Starch hydrolysis during in vitro oro-gastro-intestinal digestion using the INFOGEST protocol was analyzed and oral processing was simulated by applying two different disintegration processes (basic homogenizer, crystal balls). The bread structure, and thus the shaping method during breadmaking, significantly affected the bolus particle size during all digestion stages. The different in vitro oral processing methods affected the bolus particle sizes after the oral phase in both breads, but they affected the particle size distribution after the gastric and intestinal phase only in the case of loaf bread. Aggregates were observed in the gastric phase, which were significantly reduced in the intestinal phase. When simulated oral processing with crystal balls led to bigger particle size distribution, bread rolls presented the highest in vitro starch hydrolysis. The type of in vitro oral processing allowed discrimination of the performance of the structures of the two breads during starch hydrolysis. Overall, crumb structure significantly affected texture properties, but also had a significant impact on particle size during digestion and starch digestibility.

## 1. Introduction

Dietary guidelines recommend the consumption of carbohydrate-rich foods as an important source of nutrients. During human digestion, carbohydrate-rich foods are broken down, releasing high amounts of sugars, which have been related to metabolic diseases [1] and are the foundation for several concerns about their long-term consumption [2,3]. However, there is not a direct relationship between food chemical composition and these effects on health because alterations in food matrix structures lead to differences in nutrient bioavailability, rates of absorption and post-prandial outcomes that might modify their potential health risks [4]. Additionally, the breakdown of the food matrix during the digestion process affects the rate at which foods are digested [5]. Therefore, clear attention should be given to the food matrix structure as well as to the food digestive process in order to understand how to control the glycemic index of carbohydrate-rich foods.

Bread represents one of the principal components of the human diet worldwide. Generally, bread matrix structure has been described as an open-cell foam consisting of highly connected pores. This porosity causes not only the characteristic bread structure but also its classification as a high glycemic index product [6]. However, modifications in bread-making process induce quality variations, including texture changes [7], and the relationship between wheat bread structure and the postprandial metabolic response has been established [8]. Eelderink et al. [8] reported that a more compact bread structure, caused by different processing conditions, resulted in a healthier bread. In addition, a review conducted by Björzack et al. [9] on the glycemic index of wheat bread stated that sourdough fermentation, reduced bread volume or kneading time, and long fermentations resulted in a reduction in glycemic index. From a digestive point of view, food structure can significantly affect the digestibility by modifying the degradation degree [5]. 

In vitro digestion systems have been more than adequate for assessing the rate of carbohydrate digestion and absorption, namely the glycemic index [9]. These methods are useful for studying gastrointestinal food degradation without human intervention and provide an alternative to in vivo methods [10]. Because of that, several in vitro systems simulating gastrointestinal digestion have been developed and improved [11]. However, many of the digestion protocols vary depending on the study, so results are often not easily comparable. To overcome this, the INFOGEST cost action recently proposed a consensus document describing a realistic digestion system to simulate food degradation during digestion [12]. This method represents a valuable tool for determining the glycemic index. Nevertheless, the evaluation of in vitro food degradation is still complicated because both disintegration of food to smaller particles and lubrication of food mass with simulated fluid should be considered. Previous studies have examined oral bread disintegration using different strategies [5], such as cutting, cut-and-pestle, blending and grinding, with the former two methods providing similar physical characteristics to in vivo mastication [5]. Although mastication is a very complex stage, comprising food breakdown and its simultaneous lubrication with saliva, these methods have provided a representative physical measure of the in vivo situation. Apart from this first stage breakdown, the disintegration of swallowed food continues during digestion. However, bread disintegration in the stomach and intestine using an in vitro model has scarcely been studied.

In the framework of deepening the present knowledge of the influence of bread structure on the bread glycemic index, two different breads were produced using the same ingredients but varying the shaping process. Resulting breads were subjected to an in vitro oro-gastro-intestinal digestion, by applying different methods to the disintegrating breads, to determine the impact of the bolus particle size distribution and its influence on the glycemic index. Simultaneously, the textural parameters of the bread were evaluated in order to determine the texture effect on bolus disintegration during digestion, and on the glycemic index.

## 2. Materials and Methods

### 2.1. Materials

White wheat flour was purchased from Harinera La Meta S.A (Lleida, Spain). Dry baker’s yeast was provided by Lesaffre Group (Valladolid, Spain). The rest of the ingredients were acquired from the local market.

Type VI-B α-amylase from porcine pancreas (EC 3.2.1.1), mucin from porcine stomach Type II (EC 282.010.7), pepsin from porcine gastric mucosa (EC 3.4.23.1), pancreatin from porcine pancreas (EC 232.468.9), bile salts and 3,5-dinitrosalicylic acid (DNS) were purchased from Sigma Aldrich (Sigma Chemical, St. Louis, USA). Solutions and standards were prepared by using deionized water.

### 2.2. Bread Preparation

Bread preparation was based on a simple recipe (100% wheat flour, 56.1% water, 1% dry baker’s yeast and 1.5% salt). The amount of water used was previously determined and was that required to yield a maximum dough consistency of 1.1 Nm. The mixture was kneaded for 12 min in a mixer (Mahot Labo 25, VMI, Montaigu Vendée, France) at a high speed. After that, the dough was divided into 200 g pieces that were subjected to that were subjected to either automatic sheeting and rolling (L) (Ciberpan, Castellón, Spain) and placed into cardboard pans, or bowling (B) to form different matrix structures. The resulting breads were leavened in a proofing chamber (Salva, Gipuzkoa, Spain) at 30 °C for 60 min and were then baked in an electric oven (F106, FM Industrial, Córdoba, Spain) at 185 °C for 25 min. After baking, the breads were cooled down at room temperature for 60 min. The breads were characterized one hour after baking, packed in polyethylene bags and stored at −18 °C for further analysis. Baking was performed by two independent trials.

### 2.3. Bread Characterization

Quality parameters including moisture, crumb texture profile analysis (TPA) and crumb image analysis were assayed according to Matos and Rosell [13]. Moisture was determined following the ICC (International Association for Cereal Science and Technology) standard method (ICC 110/1) [14]. Hardness, springiness, cohesiveness, chewiness and resilience parameters were recorded from the TA.XT-Plus Texture Analysis (Stable Micro Systems Ltd., Godalming, UK) equipped with a 5 kg load cell. Parameters were measured in 10 mm central vertical slices of the resulting breads with the crust removed. During the test, the center of the crumb was double compressed with a 25 mm aluminum cylindrical probe at a crosshead speed of 1 mm/s and 30 s gap between compressions. Data from five slices per bread were averaged.

Bread crumb structure was analyzed using an image analysis system as previously described Morreale et al. [15]. Data acquired from the crumb structure analysis (slice 2D area (cm^2^) and surface porosity (%)) were used for comparing the different breads. 

### 2.4. In Vitro Oro-Gastro-Intestinal Digestion

Before digestion, bread samples were defrosted and then subjected to successive oral, gastric and intestinal digestion following the standardized static digestion method developed by Minekus et al. [12]. The selection of this protocol was based on physiologically relevant conditions.

Five grams of bread crumbs were added to 5 mL of simulated salivary fluid (SSF), containing 0.5 mL of α-amylase solution (1500 U/mL), 0.05 mL of mucin solution (0.006 g/mL) and 0.025 mL of 0.3 M CaCl_2_ in SSF (prewarmed at 37 °C). Then, the mixture was subjected to two different disintegrating methods, to simulate oral processing, in order to obtain boluses with different degrees of fracturability. The first method (P) was accomplish by using an Ultra Turrax T18 basic homogenizer (IKA-Werke GmbH and Co. KG, Staufen, Germany), while the second one (B) included an Ultra Turrax Tube Drive with crystal balls (IKA-Werke GmbH and Co. KG, Staufen, Germany). For each method, the mix was stirred for 2 min at 37 °C. Gastric digestion was immediately performed by adding 7.5 mL of simulated gastric fluid (SGF) containing 1.6 mL of pepsin solution (25,000 U/mL), 0.005 mL of 0.3 M CaCl_2_ and enough volume of 8 M HCl to adjust the pH to 3. The mix was then digested in a shaker incubator SKI 4 (ARGO Lab, Carpi, Italy) at 37 °C under constant stirring at 150 rpm. After 2 h of gastric digestion, intestinal digestion was simulated by the addition of 11 mL of simulated intestinal fluid (SIF) containing 5 mL of pancreatic solution (800 U/mL) and 2.5 mL of 160 mM bile extract solution and 0.04 mL of 0.3 M CaCl_2_. The pH was adjusted to 7.0 and then, the final mix was digested for 3 h at 37 °C under constant stirring at 150 rpm.

Different aliquots were withdrawn from the reaction vessel at different intervals of each phase of digestion. All aliquots (400 µL) were immediately mixed with 400 µL ethanol (96%) in order to stop enzyme hydrolysis. Then, the aliquots were centrifuged at 10,000 × *g* and 4 °C for 5 min. The pellet was washed with 200 µL ethanol (50%). The supernatants were collected and stored together at −20 °C until further use.

### 2.5. Reducing Sugars Released and In Vitro Starch Digestibility 

Aliquots from intestinal digestion were employed to determine the concentration of released reducing sugar using the DNS method. The amounts of reducing sugars were measured spectrophotometrically (λ = 540 nm) using an Epoch microplate reader (Biotek Instruments, Winooski, VT, USA). The released reducing sugars were converted into starch and expressed as glucose (mg) × 0.9.

The amount of hydrolyzed starch was plotted against the digestion time (min) after fitting experimental data to a first-order equation [16]:

(1) $$C=C_{\infty }\;\left(1-e^{-\mathrm{kt}}\right)$$

*C* is the percentage of starch hydrolyzed at time *t*, *C*_∞_ is the equilibrium percentage of starch hydrolyzed after 180 min, *k* is the kinetic constant and *t* is the time (min). The hydrolysis index (HI) was obtained by dividing the area under the hydrolysis curve (0–180 min) of the sample by the area of a standard material (white bread) over the same period of time. The expected glycemic index (*eGI*) was calculated using the equation eGI=39.21+0.803 HI90 [16].

### 2.6. Particle Size Distribution of the Bolus during In Vitro Digestion

The particle size in the in vitro digestion fractions was observed using a digital camera (EVOCam, Vision Engineering Ltd, Surrey, England). Prior to observation, the bolus samples were diluted with 150 mL of glycerol in Petri dishes (9 cm diameter) at room temperature [17]. Samples were examined with a magnification of 3.78×. Then, high-resolution images of the particles were acquired and the particle size distribution was analyzed using the image analysis program (ImageJ, UTHSCSA Image Tool software, Barcelona, Spain) and NIS-Elements (Nikon Instruments Inc., Tokyo, Japan) software. Images were saved as an 8-bit tiff format and the MidGrey auto local thresholding was subsequently applied with the ImageJ. Crumbs were analyzed with the NIS-Elements software, removing particles with a mean intensity value less than 150. The scale was initially set using the relationship between pixels and known distance, and then, a box plot displaying the distribution of particle size (corresponding to the particle length) was built.

### 2.7. Statistical Analyses

Experimental data were subjected to an analysis of variance (ANOVA) and values were expressed as average ± confidence interval of at least two individual measurements, using Statgraphics Centurion XV (Statistical Graphics Corporation, Rockville, MD, USA). Fisher’s least significant differences (LSD) test was used to describe means with 95% confidence. Pearson correlation coefficient (*r*) and *P*-value were used to indicate correlations. Differences of *P* < 0.05 were considered significant. All measurements were performed at least in duplicate. For the particle size distribution analysis, the non-parametric Kruskal–Wallis test was applied to identify possible significant differences between population medians. Furthermore, the data was investigated by multivariate data analysis (principle component analysis (PCA)) with R software version 3.5.0. to determine the differences among the samples.

## 3. Results 

### 3.1. Variation of Bread Structure as a Consequence of Changes in the Breadmaking Process

**Figure 1:**
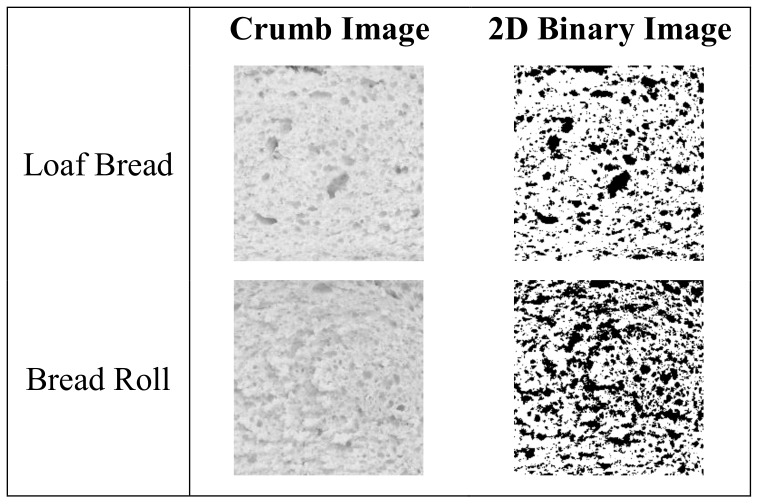
Crumb image of wheat breads shaped to conform to the requirements of the loaf bread and bread roll.

A simple recipe for wheat bread was used to obtain the dough pieces, which were shaped to conform the requirements of the loaf bread (L) or bread roll (B). The effect of the shaping process on the technological properties of the end-breads are summarized in Table 1. The statistical analysis revealed that the method by which dough pieces were shaped induced significant (*P* < 0.05) variations in moisture, volume (slice 2D area), texture, and crumb morphology of bread. The slice 2D area was significantly lower in L bread than in B bread, which might be related to the dough sheeting that forces the partial release of the carbon dioxide produced during bulk leavening. The images of the crumb bread sections can be observed in Figure 1. Parameters derived from the image analysis are summarized in Table 1.

The crumb morphology of the end-breads was significantly (*P* < 0.05) influenced by the method applied to shape the dough (Table 1). This agrees with previous research reported by Gao et al. [7], who showed alterations in the matrix structure due to changes in the process conditions. The authors produced three different types of bread (baguette, baked bread and steamed bread) with different structures by changing the mixing and proofing conditions, as well as the proofing and baking times. In the current study, resulting pieces of bread exhibited a highly porous crumb structure with open pores. The loaf crumb had lower porosity and larger average cell area, in opposition to the roll performance. During shaping, different pressures were applied to the doughs in order to obtain the two types of breads. It has been reported that doughs subjected to different magnitudes of force can undergo modifications in the structure of gluten, which is related to the capacity of the mass to retain gas bubbles [18]. The distinct cellular structure can be attributed to the different pressures applied during dough shaping, which altered the gas bubble structures.

Owing to the different crumb cell structures, different texture properties were also expected. The variations in the shaping process significantly (*P* < 0.05) influenced the crumb hardness, springiness, cohesiveness, chewiness and resilience (Table 1). Loaf bread had softer crumbs with lower chewiness and springiness, but higher cohesiveness and resilience, indicating that its structure was rapidly recovered after compression. According to Cauvain [18], doughs with different gas retention provide breads with changes in crumb texture, which agrees with the previous observations described in the crumb morphology. Therefore, variations in the compact degree of bread structure could be obtained by only modifying the shaping of the dough that, in turn, led to changes in texture parameters.

### 3.2. Bolus Particle Size throughout In Vitro Digestion

**Figure 2:**
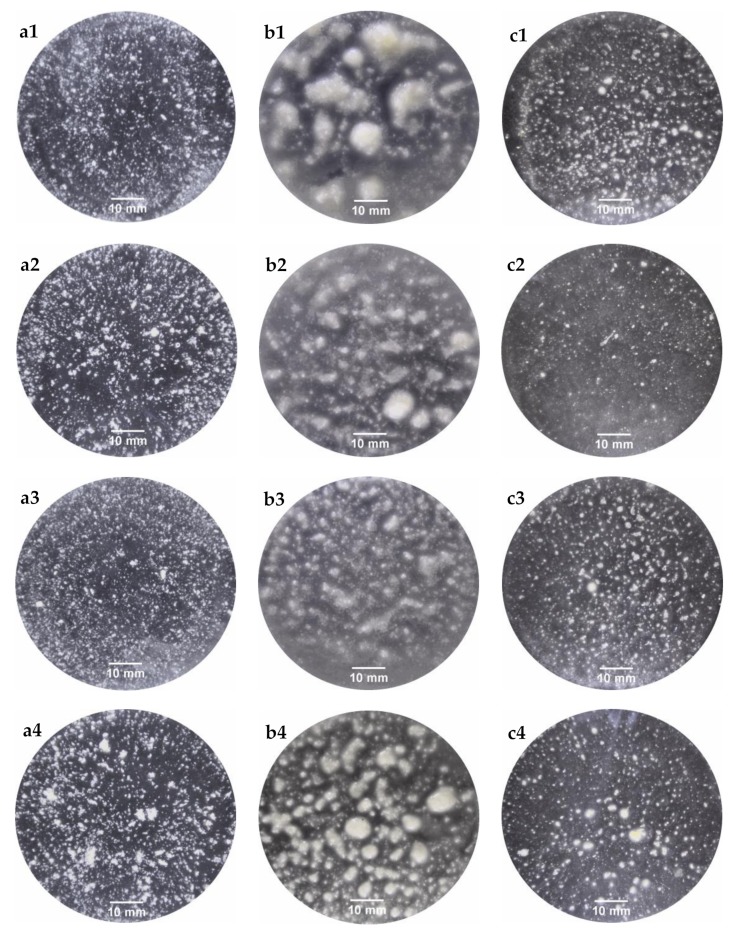
Representative images of bolus particles obtained after oral (**a**), gastric (**b**) and intestinal (**c**) in vitro digestion. Boluses were obtained from loaf bread (**1**,**2**) or bread rolls (**3**,**4**) using Ultra Turrax (**1**,**3**) or Ultra Turrax with crystal balls (**2**,**4**) as simulated oral processing methods.

Figure 2 shows the visual appearance of L and B bread bolus particles at the final stages of the oral (Figure 2a), gastric (Figure 2b) and intestinal (Figure 2c) phase using a basic Ultra Turrax homogenizer (P) (Figure 2, letters followed by 1 or 3) or Ultra Turrax with crystal balls (B) (Figure 2, letters followed by 2 or 4) as simulated oral processing methods.

**Figure 3:**
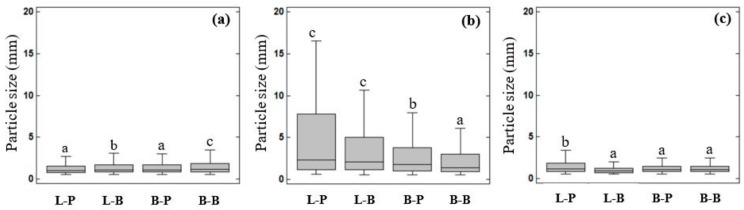
Bolus particle size obtained after the oral (**a**), gastric (**b**) and intestinal (**c**) phase of in vitro digestion. Sample names describe bread type (L-loaf, B-roll bread) followed by letters describing the simulated oral processing method applied (P Ultra Turrax and B Ultra Turrax with crystal balls). Letters on the bars indicate significant differences (*P* < 0.05).

The bolus particles displayed different visual aspects that changed over the in vitro digestion. To clearly represent the particle size distribution of each bolus, analysis of the images was carried out to obtain the particle lengths and a boxplot was constructed giving the maximum and minimum values of the particle length for each bolus, as well as the upper and lower quartiles and the median values (Figure 3). The ANOVA analysis indicated that the method used to shape the dough significantly affected the particle size distribution. The simulated oral processing methods significantly affected the particle size distribution obtained after the oral phase in both breads, whereas the particle size distribution after the gastric and intestinal phase was significantly affected only in the case of loaf bread.

The first step of food digestion is oral processing. In the oral cavity, the original food structure is transformed by the action of teeth and the tongue [19], leading to the formation of a reduced structure that can be safely swallowed [20]. In this regard, L-P and B-P pieces of bread were broken down into smaller particles, with a median value of 1.03 mm. A larger particle size was found in the B-B sample (median value of 1.10 mm), followed by the L-B sample (median value of 1.06 mm) (Figure 3a). The mean area of the particles for L-P, L-B, B-P, B-B were 0.87, 1.15, 0.77 and 1.56 mm^2^, respectively. These results were lower than those (20–69 mm^2^) previously reported by Gao et al. [5], who used different simulated oral processing methods. Nevertheless, the majority of the bolus particles (~90%) that they obtained, with all the tested in vitro methods, was larger than the 2 mm recommended by the standardized method [12]. It has been also described that after chewing 10 different natural foods, the median particle size (*d_50_*) ranged from 0.8 to 3.04 mm, indicating their different fracturability [21]. Therefore, both methods used in the present study could be adequate tools to disintegrate foods when using in vitro methods. Once the food is swallowed, it is transported to the stomach. Unlike that reported by other authors [3,22,23], part of the individual oral particles agglomerated during the gastric phase (Figure 2b). As a consequence, an increase in the particle size was observed, along with a wider size distribution in all samples. Among them, the L-P sample exhibited a larger particle size (median value of 2.27 mm), while the B-B sample displayed the lowest one (median value of 1.37 mm). In the stomach cavity, food comes in contact with gastric juice, affecting its physicochemical properties, such as size, surface charge and agglomeration state [24]. To properly simulate the gastric environment, the commonly suggested medium possesses a pH around 3. In acidic medium, mucin particles form large aggregated chains [25]. Based on that, the agglomerates and, in turn, the increase in particle size could be attributed to the presence of mucin in the medium. Food digestion ends in the intestinal cavity. In the current study, the intestinal bolus particles appeared to become smaller with more homogeneous sizes (Figure 2c), with medians ranging from 0.87 to 1.16 mm (Figure 3c). It is worth noting at this point that mucin particles disperse as a function of pH [25]. Therefore, the results obtained in the intestinal phase reinforce the suggestions mentioned for the gastric stage.

### 3.3. In Vitro Digestion and Expected Glycemic Index

The glycemic index has been employed as a reference tool to classify the rate of carbohydrate digestion and absorption of foods [2,16,26]. Therefore, the glycemic index of L and B bread based on the application of a simulated small intestinal in vitro digestion system was measured. In addition, primary and secondary parameters derived from intestinal digestion were analyzed (Table 2).

**Figure 4:**
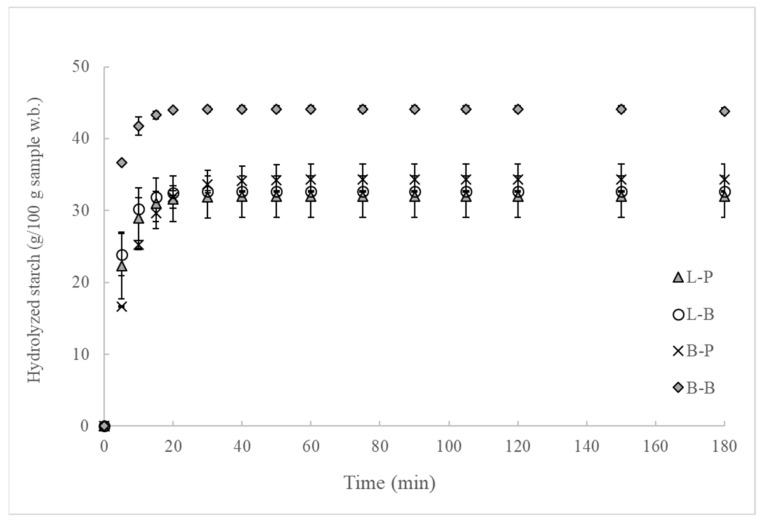
Effect of bread structure and simulated oral processing method on starch hydrolysis pattern. Sample names describe the shaping method used (L rolling mill process and B balling process) followed by letters describing the simulated oral processing method applied (P Ultra Turrax and B Ultra Turrax with crystal balls).

In line with previous reports [26,27,28], the quantification of glucose released increased linearly during the first 20 min of intestinal digestion (Figure 4), and the kinetic constant (*k*) for the amylolysis was not significantly affected by the shaping process or simulated oral processing method. After that, a slow release of glucose was observed, reaching a plateau after 40 min of intestinal digestion. 

The statistical analysis indicated that the shaping method had a significant (*P* < 0.05) effect on the equilibrium concentration of hydrolyzed starch (*C*_∞_), the area under the hydrolysis curve after 180 min, hydrolysis index (HI) and estimated glycemic index (*eGI*) parameters. Results obtained distinguished two different groups. The first group contained the L-P, L-B and B-P bread, which displayed similar values for these parameters. While the second group, containing the B-B sample, showed higher values for the previously mentioned parameters. A deeper statistical analysis carried out for each of the in vitro oral processing methods revealed that the bread structure only exerted a significant influence when the simulated oral processing method was B. Therefore, results indicated that the method used to simulate the oral processing process plays a fundamental role in in vitro digestion. This makes the settings defined for carrying out in vitro oral systems that closely follow the conditions of in vivo mastication deeply relevant.

### 3.4. Multivariable Analysis

**Figure 5:**
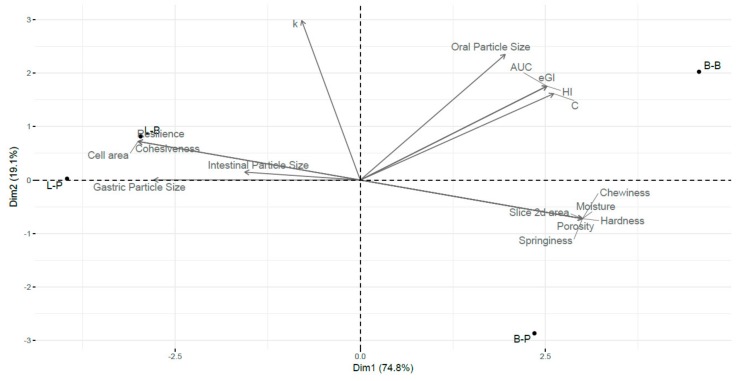
Score and loading biplot Dimension 1 × Dimension 2 of samples and variables obtained by principal component analysis (PCA). Samples are labelled as in the text.

The PCA created from technological characteristics, as well as the digestion parameters measured, was used to summarize the relationship between the L and B bread digested with different simulated oral processing methods, providing easier visualization (Figure 5). PC1 accounted for 74.8% of the determined variances mainly explaining the variation in textural properties. Whereas the second PC accounted for 19.1% of the determined variances, representing principally the digestion parameters.

The loadings indicated a weak correlation between the parameters associated with bread structure and glycemic index. Nevertheless, the digestion parameters were strongly related to the oral particle size. In addition, it is important to note the negative relationships observed between structural bread parameters (porosity, hardness, chewiness, springiness, volume) and moisture, with gastric and intestinal particle size distribution, with exception of resilience and cohesiveness. It is assumed that the greater bolus disintegration, the higher glycemic index obtained, which was observed in the PCA, although the correlation obtained with gastric or intestinal particle size distribution and glycemic index was not significant.

The PCA clearly discriminated between loaf bread and bread roll, particularly along PC 1. This was a result of the different matrix structures obtained by applying different shaping methods. While L bread was found to the left of the plot, B bread was at the right side, reflecting its higher moisture, volume, hardness and porosity. Similar results were obtained by Bornhorst and Singh [23] who observed that low moisture content in the bread promotes a large amount of gastric fluid absorption. The authors also reported that bolus disintegration varied depending on the bread structure, being faster in the bread with the highest hardness. In fact, B bread that showed higher hardness might be quickly disintegrated, giving higher starch hydrolysis. Across PC2, the samples were split between the different simulated oral processing methods used. Hence, the samples digested with Ultra Turrax could be found in the bottom half, which might be due to the low oral particle size distribution and lower glycemic index. Therefore, PCA allowed discrimination between the two crumb bread structures based on their digestibility and physical properties, indicating that breads with higher moisture, porosity, volume and hardness gave higher oral particle size and higher starch digestibility.

## 4. Conclusions

The shaping step in breadmaking played a significant role in bread structure, obtaining breads with different morphological and textural parameters. The different crumb bread structures obtained and the in vitro oral processing method used, affected the bolus behavior along the in vitro digestion, achieving different particle sizes.

Starch hydrolysis through the in vitro digestion of bread showed a typical trend and it was affected by the bread structure. Bread roll masticated with Ultra turrax with crystal balls showed higher starch hydrolysis, obtaining higher e*GI* values, but no differences were observed when oral disintegration was carried out with a basic homogenizer. Therefore, the type of oral processing method applied to fractionate bread might allow discrimination of the performance of two bread structures during starch hydrolysis.

Overall, this study indicated that bread structure and simulated oral processing play an important role in bread digestion. Therefore, a gastrointestinal digestion analysis is essential for considering the structure of the food to be digested and the simulated method of oral processing that is carried out.

## Figures and Tables

**Table 1:** Moisture, morphological and texture parameters of the wheat bread crumbs.

	Loaf Bread (L)	Bread Roll (B)
**Moisture (%)**	28.18 ± 2.84 ^a^	36.73 ± 3.28 ^b^
**Slice 2D Area (cm^2^)**	30.86 ± 0.68 ^a^	42.06 ± 1.77 ^b^
**Cell area (mm^2^)**	0.26 ± 0.01 ^b^	0.23 ± 0.01 ^a^
**Porosity (%)**	24.38 ± 1.67 ^a^	33.88 ± 2.06 ^b^
**Hardness (N)**	1.18 ± 0.10 ^a^	2.69 ± 0.14 ^b^
**Cohesiveness**	0.95 ± 0.02 ^b^	0.86 ± 0.03 ^a^
**Chewiness**	1.02 ± 0.02 ^a^	2.74 ± 0.79 ^b^
**Resilience**	0.54 ± 0.02 ^b^	0.43 ± 0.04 ^a^
**Springiness**	0.85 ± 0.08 ^a^	0.94 ± 0.04 ^b^

Means within the same row denoted by different superscript letters differ significantly (*P* < 0.05).

**Table 2:** Kinetic constant (*k*), equilibrium concentration (*C*_∞_), area under the hydrolysis curve after 180 min (AUC), hydrolysis index (HI) and estimated glycemic index (*eGI*) for loaf bread (L) and bread rolls (B) subjected to two different simulating oral processing methods, with Ultra Turrax (P) or crystal balls (B).

Shaping Method	Oral Processing Method	*k*	C_∞_^A^	AUC	HI	eGI ^B^
L	P	0.24 ± 0.05	31.94 ± 2.88 ^a^	5600 ± 532 ^a^	70.14 ± 6.67 ^a^	64.86 ± 2.31 ^a^
	B	0.27 ± 0.06	32.66 ± 0.12 ^a^	5736 ± 48 ^a^	71.85 ± 0.6 ^a^	65.44 ± 0.10 ^a^
B	P	0.13 ± 0.01	34.31 ± 2.18 ^a^	5906 ± 355 ^a^	73.98 ± 4.45 ^a^	66.76 ± 1.75 ^a^
	B	0.27 ± 0.14	44.15 ± 0.42 ^b^	7712 ± 42 ^b^	96.6 ± 0.52 ^b^	74.66 ± 0.34 ^b^
*P*-value	Shaping method	0.3880	0.0316	0.0436	0.0436	0.0317
	Oral processing method	0.2218	0.0738	0.0771	0.0771	0.0712

Values followed by different letters within a column are significantly different (*P* ≤ 0.05). ^A^ *C*_∞_ and *k* were determined by the equation, *C* = *C*_∞_(1 − e^−*kt*^). ^B^ *eGI* was calculated from the equation proposed by Goñi et al. [16].
